# Cancer cell: using inflammation to invade the host

**DOI:** 10.1186/1476-4598-6-29

**Published:** 2007-04-16

**Authors:** José-Ignacio Arias, María-Angeles Aller, Jaime Arias

**Affiliations:** 1 General Surgery Unit, Monte Naranco Hospital, Oviedo, Asturias, Spain; 2 Surgery I Department, School of Medicine, Complutense University of Madrid, Spain

## Abstract

**Background:**

Inflammation is increasingly recognized as an important component of tumorigenesis, although the mechanisms involved are not fully characterized. The invasive capacity of cancers is reflected in the classic metastatic cascade: tumor (T), node (N) and metastasis (M). However, this staging system for cancer would also have a tumoral biological significance.

**Presentation of the hypothesis:**

To integrate the mechanisms that control the inflammatory response in the actual staging system of cancer. It is considered that in both processes of inflammation and cancer, three successive phenotypes are presented that represent the expression of trophic functional systems of increasing metabolic complexity for using oxygen.

**Testing the hypothesis:**

While a malignant tumor develops it express phenotypes that also share the inflammatory response such as: an ischemic phenotype (anoxic-hypoxic), a leukocytic phenotype with anaerobic glycolysis and migration, and an angiogenic phenotype with hyperactivity of glycolytic enzymes, tumor proliferation and metastasis, and cachexia of the host. The increasing metabolic complexity of the tumor cell to use oxygen allows for it to be released, migrate and proliferate, thus creating structures of growing complexity.

**Implication of the hypothesis:**

One aim of cancer gene therapy could be the induction of oxidative phosphorylation, the last metabolic step required by inflammation in order to differentiate the tissue that it produces.

## Background

The link between inflammation and the development of cancer has been recognized since 1863, when Rudolf Virchow discovered leukocytes in neoplastic tissues and made the first relation between inflammation and cancer [1]. Since then, a number of cancers have been linked to inflammatory origins [2] and in many cases it has been considered how the tumor microenvironment highly resembles an inflammatory site [3,4]. Nowadays, the causal relationship between inflammation and cancer is widely accepted.

Inflammation is increasingly recognized as an important component of tumorigenesis, although the mechanisms involved are not fully characterized [4-6].

Tumors can be noninvasive or benign, because they are cured easily by simple removal, and invasive -also called malignant or cancers that invariably kill their host if untreated [7]. This invasive ability of cancers is reflected in the classic metastatic cascade, which is staged according to the volume of the primary tumor and its depth of invasion (T stage), the number and the volume of occupied lymph nodes and the invasion through their capsule (N stage), as well as the presence of distant metastases (M stage) [7].

However, a staging system for cancer should fulfill two important characteristics: the first of these would be to ensure a common terminology for cancer that can be understood by clinicians in all specialities and the second is that this system, for example, the tumor node, and metastastis one (TNM) should be continually submitted to critical evaluation and change when clinically indicated [8].

One requirement for both conditions to be fulfilled would be that the staging system would also have a tumoral biological significance. Therefore, it should be kept in mind that while a malignant tumor develops, it can express phenotypes that also share the inflammatory response.

According to Elias Zerhouni, as science grows more complex, it is also converging on a set of unifying principles that link apparently disparate diseases through common biological pathways and therapeutic approaches [9]. Thus research tactics and strategies may become very similar across diseases [9,10]. In this way, to integrate the mechanisms that govern the inflammatory response with the actual staging system of cancer, could enrichen the pathogenic knowledgement of the malignant tumor.

## Presentation of the hypothesis

The successive pathophysiological mechanisms that develop in the interstititum of tissues when they undergo acute post-traumatic inflammation are considered increasingly complex trophic functional systems for using oxygen [11-13].

The nervous or immediate functional system presents ischemia-revascularization and edema, which favor nutrition by diffusion through injured tissue. This trophic mechanism has a low energy requirement that does not require oxygen (ischemia) or in which oxygen is not correctly used, with the subsequent development of reactive oxigen species (reperfusion) [11,13].

The immune or intermediate functional system produces coagulation and infiltration of the injured tissue by inflammatory cells, especially by leukocytes. Hence, extracelullar digestion, by enzyme release (fermentation) and intracellular digestion by phagocytosis may be associated with a hypothetical trophic capacity of the neighbouring cells. Improper use of oxygen persists in this immune phase. Activated phagocytes would require anaerobic glycolysis as the main source of ATP for their functions. During this phase, lymphatic circulation plays a major role and macrophages and dendritic cells migrate to lymph nodes [11,13].

Finally, the endocrine functional system facilitates the arrival of oxygen, transported by red blood cells and capillaries. Angiogenesis characterizes this last phase of the inflammatory response, and therefore nutrition mediated by the blood capillaries is established. Oxygen and oxidative metabolism are an excelent combination by which the cells can obtain an abundant energy supply in tissue repair by epithelial regeneration or wound healing [11-14].

The expression of the nervous, immune and endocrine functional systems during the inflammatory response makes it possible to differentiate three successive phases, which progress from ischemia, through a metabolism that is characterized by defective oxygen use (reperfusion, oxidative burst and heat hyperproduction), up to an oxidative metabolism (oxidative phosphorylation) with the correct use of oxygen that produces usable energy. Thus, it is also tempting to speculate on whether the body reproduces the successive stages from which life passes from its origin without oxygen until it develops an effective, although costly, system for the use of oxygen every time we suffer acute inflammation [11-13,15].

The sequence in the expression of progressively more elaborated and complex nutritional systems could hypothetically be considered the essence of the inflammation, regardless of what its etiology or localization may be. Hence, the incidence of harmful influences during their evolution could involve regressing to the most primitive trophic stages, in which nutrition by diffusion (nervous phase) takes place. This is simpler, but also less costly and facilitates temporary survival until a more favorable enviroment makes it possible to initiate more complex nutritional methods (immune and endocrine phases) [11-13,15].

If this hypothesis about inflammation is applied to tumor development, it could be accepted that both benign and malignant tumors also use the three functional systems, nervous, immune and endocrine, already mentioned above, for their evolution.

Thus, benign tumor cells seem to be able to induce the inflammatory response in the host and collaborate in establishing the tumor through a process called desmoplasia [7]. Essentially, all the elements that constitute the inflammatory response participate in the "host reaction" which could, therefore, have a trophic purpose for the tumor cells [16].

However, malignant tumor cells with their invasive capacity, reflected in the classic metastasic cascade T-N-M [17], seem to have the new ability to express the inflammatory response rather than induce it in the host. In this hypothetical circumstance, the successive phases that have been described in the inflammatory response would be expressed by malignant tumor cells and would express their evolution with invasion and metastasis [16,18].

## Testing the hypothesis

The understanding of the pathogenesis and progression of cancer requires the establishment of the altered genetic/metabolic factors that are essential to the development, growth and proliferation of malignant cells [19]. This new frontier of cancer research requires the appropiate marriage of genetic/proteomic studies or the geneticist approach with the biochemical/metabolic cellular studies or the biochemical approach [19].

With regards to the geneticist approach, in many cancers, a stem cell tumor model probably takes place [20]. Early cancer stem cells derived from normal stem cells, through a process termed "pretumor progression", or the epithelial cancer stem cells, are susceptible to accumulate multiple oncogenic changes that acquire the capacity for long-term proliferation and give rise to tumors [21].

**Figure 1:**
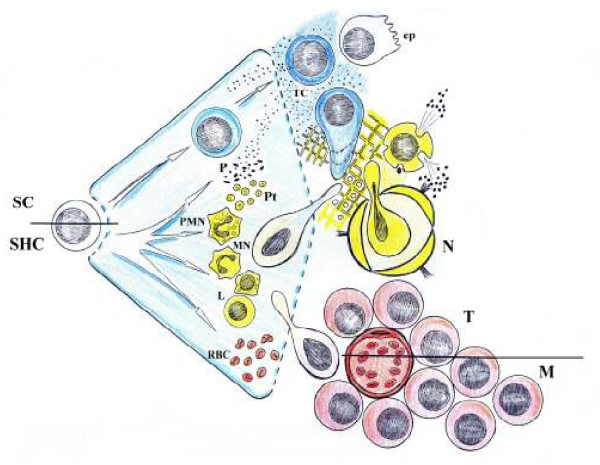
Using by the malignant tumor cell of the mechanisms involved in the inflammatory response. a : **Ischemicphenotype**. Oxidative stress is produced from the anoxia until the beginning of the oxygenation (hypoxia) and the tumor cell becomes independent (autocrine) and acquires motility. In this phase nutrition by diffusion stands out. b: **Leukocytic phenotype**. A provisional matrix is developed by hypercoagulation. The hyperproduction of enzymes, chemokines and cytokines induce the lymphatic migration (paracrine), as well as the uptake of nutrients by the cancer cell. c : **Angiogenic phenotype**. Angiogenesis makes it possible that a number of host substances, including hormones (endocrine) would be used by the tumor cell in its own metabolism. This fact favor its excessive growth and the metabolic manipulation of the host inducing cachexia. SC: Stem cell; SHC: Stem hematopoietic cell; P: plasma; Pt: Platelets. PMN: Polimorphonuclear neutrophils; MN: Monocite; L: Limphocyte; RBC: red blood cells; TC: Tumor cell; ep: epithelial cell ; N: node; T: Tumor, M: Metastasis.

With regards to the biochemist approach, metabolic transformations of malignant cells are essential to the development and progression of all cancers [19]. Owing to the plasticity of cancer stem cells [20-22] it should be born in mind that while a malignant tumor develops, it can express phenotypes that also share the inflammatory response such as: an ischemic phenotype (hypoxic) [23] (see Figure 1), a pro-inflammatory gene expression with adoption of a leukocytic phenotype [5,16] and migration to the regional lymph nodes [24] and, finally, an angiogenic phenotype [25,26]. It has already been propposed that these phenotypes represent the expression of trophic functional systems of increasing metabolic complexity in the inflammatory response [11-13]. Their expression by cancer cells could have a similar significance.

In this hypothetical circumstance, malignant tumor cells, could adopt an inflammatory-like phenotype that evolves in three hypothetical functional phases, of increasing metabolic complexity, which would have also a trophic significance [16,18].

## Evolutive phases of cancer cells

The three inflammatory phenotypes hypothetically expressed by malignant tumor cells during the host invasion, could help to integrate the etiopathogenic factors that the cancer disease has. These inflammatory phenotypes would associate the genetic factors (oncogenes, mutations) responsible for cancer with the metabolic factors (celullar intermediary metabolism) [19].

Owing to the plasticity of cancer stem cells [27], these could adopt an inflammatory-like phenotype that evolves in three hypothetical functional phases with trophic significance [16,18].

In the first or nervous phase characterized by the ischemia-reperfusion phenomenon, the tumor cells undergo oxidative stress, become independent and mobile. In this phase, malignant tumor cells are nourished by diffusion [16,18]. Tumor cells seem to adopt an ischemic (anoxic-hypoxic) phenotype [23] (see Figure 1), namely, when tumor cells come into contact with oxygen, first they undergo a process of reoxigenation, with oxidative stress and edema [18]. The hypoxic microenvironment occurs very early during tumor development, when the tumor reaches approximately 2–3 mm in diameter [28,29]. The transcriptional response to hypoxia relies on multi-protein complexes to regulate several transcription factors. The most well studied mechanism identified in this process is an interaction of a family of transcription factors, called hypoxia-inducible factors (HIF) with a cis-acting element, called the hypoxia-responsive element, located in regulatory regions of target genes. The most widely studied HIF heterodimer is HIF-1, that is the principal regulator of the hypoxic response in most mammalian cells. HIF-1 enhances the expression of hypoxia-responsive genes and, therefore, allows improved cell survival in conditions of limited oxygen availability [23,28,29]. Otto Warburg described in 1956 the tumor's increased dependence upon fermentation or anaerobic glycolysis [30]. Today, it is considered that a specific transcriptional program, initiated by HIF-1, increases the expression of glycolytic enzymes [29,31,32]. Therefore, activation of glycolytic genes and metabolism or glycolytic tumoral switch is considered a metabolic adaptation to hypoxia through increased conversion of glucose to pyruvic acid and, subsequently, to lactic acid [31,32]. Hypoxic tumor cells, through the glycolytic switch as the main source of ATP, also become resistant to apoptosis, present inhibition to differentiation and become more likely to migrate to less hypoxic areas [33,34]. Moreover, during these initial vascular stages of tumor growth, which is when the tumor mass is less than 0.5 cm, nutrition can be achieved by diffusion [35].

In this evolutive stage, the tumor cell could acquire motility and, therefore, an invasive ability. Malignant epithelial cells use specific integrins to allow for survival, motility and invasion [36]. Tumor cell spreading in this early evolutive stage could involve a reorganization of the actin cytoskeleton, that is mediated through integrin [36,37]. Malignant tumor cells utilize actin to produce a propulsive form of movement in which highly deformable tumor cells can travel through the tissues [37]. They do not degrade matrix but instead, go around matrix barriers and squeeze through narrow spaces by ameboid migration [36,38].

The progressive formation of reactive oxygen species (ROS) secondary to gradual oxigenation of the tumor cell would increase its oxidative and nitrosative ability. In this hypothetical situation, oxidative stress could favor a more aggressive type of host invasion [11]. As a result, during the earlier phase of tumor progression, the metabolic autonomy and invasive capacity of the tumor cell would induce their premature migration to the peripheral tissue [16]. Malignant cells are able to remain "dormant" but viable for some period of time after the host invasion [39]. It has been proposed that tumor dormancy may be due to preangiogenic micrometastasis which subsequently acquires the ability to become vascularized [35,40].

In the second or immune phase, tumor cells could produce coagulation and express a leukocytic phenotype with anaerobic glycolysis as the main source of ATP. This phenotype permits tumor cells to develop adhesive interactions with the surrounding matrix and cells, lymphatic migration and invasion of the host lymph nodes [16,18].

Hypercoagulation in cancer patients is another factor for tumor progression. Although the mechanism behind hypercoagulation in cancer patients is unclear, the main factor responsible for hypercoagulation has been considered to be cancer itself, by producing and secreting procoagulant substances accompanied by decreasing of coagulation inhibitors [41]. By hypercoagulation, cancer cells acquire a provisional matrix that favors both their survival and invasion [11,14].

Tumor cells with leukocytic phenotype can overexpress matrix metalloproteinases (MMPs). In many instances, therefore, the extensive alterations produced by MMPs in the stromal microenvironment of epithelial cancer stem cells promote tumor progression [21]. They travel more slowly than cells using ameboid migration but are more destructive [36]. However, tumoral cell nutrition could be mediated by this great extracellular digestive potential. In this hypothetical situation, the degradation of the surrounding matrix by proteases would favor nurishing the tumoral cell and, therefore, its ability to proliferate and invade. Aggressive epithelial cancer cells using this mechanism of invasión or "mesenchymal-like mechanism" are regarded as having undergone dedifferentiation through a process called epithelial-mesenchymal transition (EMT) [36,42].

Reoxigenation of hypoxic tumor cells may cause reperfusion injury with oxidative and nitrosative stress, like what occurs in the inflammatory response by the generation of ROS and nitrogen reactive species [43]. In most inflammatory responses, the actions of ROS are mediated by the IκB/nuclear factor (NF)-κB system and, this system, in turn, can be regulated by hypoxia and/or reoxygenation [23]. More specifically, the expression of inducible genes leading to the synthesis of cytokines, chemokines, cytokine and chemokine receptors, adhesion molecules and autacoids relies on transcription factors, and among the primary transcription factors, NF-κB plays a main role in the regulation of inflammatory mediators [44].

The activation of general stress-responsive transcription factors, such as NF-κB, in tumor cells [45-47], could imply their complete transition to a hypothetical phase called an immune phase, since this could be associated with pro-inflammatory gene expression. Hence, tumor cells may co-opt key mechanisms by which inflammation interfaces with cancer, to further their colonization of the host it [16,18]. Hence, the activation of NF-κB in carcinoma cells results in elevated expression of cell-cycle genes, such as cyclin D1, inhibitors of apoptosis, and proteases that promote invasión [47].

The hypothetical activation of the leukocytic phenotype in malignant tumor cells would permit these cells to fulfill functions characteristic of activated inflammatory cells (see Figure 1). For example, functions associated with neutrophils like the hyperproduction of extracellular proteases, such as MMPs and other protease enzymes that carry out a true extracellular digestion of the basement membrane and the extracellular matrix, also aid in invasiveness in the early stages of the disease [48,49]. Cancer cells can present pseudopodia formation and directional migration as well [36,42,50].

Other functions seem to correspond to a monocyte-macrophage phenotype, in the sense that tumor cells migrate to the regional lymph nodes through lymphatic capillaries [51]. It has been suggested that malignant cells may adopt normal mechanisms of lymph node homing during metastasis [24]. The adoption of an immune phenotype by cancerous cells is associated with the formation of new lymphatic vessels [41,51].

During the immune phase of tumor progression, soluble factors could push tumor cells towards a monocyte-like phenotype and induce their premature migration to peripheral tissue. Hence, the monocyte phenotype of the tumor cells would favor the homing of metastatic tumor cells to specific organs, specially those where populations of resident macrophages are abundant, i.e. lung (alveolar macrophages), liver (Kuppfer cells), brain (microglia) and bone (osteoclasts) [16]. Cancer cells may also secrete factors that cause bone marrow-derived hematopoietic progenitor cells to migrate to premetastatic sites [52]. Therefore, a premetastatic "trophic" niche may be formed by bone marrow-derived hematopoietic cells that attract tumor cells and support a devoloping metastasis [52-54]. Besides, cells with stem cell features have been identified neither in tumors nor in metastatic lesions [55]. Therefore, cells with different grades of dedifferentiation from stem cells to bone-marrow-derived hematopoietic cells seem be involved in the metastatic colonization [53].

Particularly, disseminated tumor cells in bone marrow can be detected in 20–40% of cancer patients without any clinical or histopathological signs of metastasis [56]. The particular bone marrow environment may allow these cells to survive and disseminate later into other distant organs. This "dormant stage" of disseminated tumor cells may explain why these cells are relatively resistant to chemotherapy [57].

Primary tumors and metastasis exhibit a similarity of matched upon gene expression profiling. However, the inflammatory mechanisms involved in the metastatic colonization could be considered organ-specific [52,53].

Cancer stem cells can also exhibit a lymphocytic phenotype. It has been suggested that an aberration in the apoptosis process leads to formation of the cancer stem cell from autoreactive T cells. Therefore, the resultant cancer stem cell still preserves some effector T cell functions, such as homing into sites of inflammation [22]. Through this hypothetic lymphocytic phenotype, the cancer cell could direct the metastatic colonization. With some similarity to lymphocytes [58], the tumor cell, having suffered primary activation, can migrate to effector specific sites (lung, liver) where they may adopt distinct functional properties. Thus, the metastatic colonization could be interpreted as an attempt of the tumor cell to carry out organogenesis, but due to an insufficient metabolic activity of metastastic tumor cells, this would fail.

The hypothetical adoption of an immune phenotype by the tumor cells would also imply the acquisition of a similar metabolism. In this situation, like the activated phagocytes (granulocytes, monocytes), their functions would require anaerobic glycolysis as the main source of ATP [59,60]. In this sense, it was shown that HIF-1α is essential for the upregulation of enzymes of the glycolytic pathway to supply phagocytes with sufficient levels of ATP [59]. Tumor cells express HIF-1α and upregulate glycolysis through the activation of the energy-sensing enzyme AMP-activated protein kinase (AMPk) [31,32,61]. AMP levels rise as the cellular ATP: ATP ratio declines. This rise in AMP activates AMPk [62]. Activated AMPk facilitates the "glycolytic switch", and provides a substrate-dependent growth advantage to malignant tumors [34].

As a result, in this second or immune phase, the tumor cell could express a leukocytic phenotype with anaerobic glycolysis as the main source of ATP, which permits the invasion of the host by both lymphatic migration (by lymph vessels and nodes) and hematic migration (micrometastases or preanagiogenic metastasis) [16,18].

The adoption of an immune phenotype by cancerous cells is associated with the formation of new lymphatic vessels [51]. Lymphangiogenesis is not only crucial for cancer cells to metastasize to the regional lymph nodes, but also offer the tumor the possibility to disseminate pro-inflammatory mediators in the host, which would produce a systemic inflammatory response syndrome (SIRS) [63]. SIRS, mediated in part by pro-inflammatory cytokines, plays a role in the genesis of cachexia, associated with both critical illness and chronic inflammatory diseases [64]. These cytokines are further thought to induce an acute phase protein response (ARP) and produce the alterations in the metabolism of lipids, proteins and carbohydrates identified as crucial markers of acute inflammation in states of malignancy and critical illness [63,64].

Finally, in a third or endocrine phase, it has been proposed that the tumor cell can be nourished by capillaries, which are present due to angiogenesis. In this phase, the tumor cell also acquires the capacity to proliferate and cause cachexia in the host [16,18]. Angiogenesis characterizes this last phase of cancer evolution and permits numerous substances, including hormones, to be transported by the blood (see Figure 1). For this reason the term "endocrine" seems appropiate for this evolutive phase.

Angiogenesis requires migration of endothelial cells into the intersticial space with the subsequent proliferation and differentiation into capillaries [18]. Tumors induce angiogenesis by activating tumor stroma cells, by releasing angiogenic factors from the extracellular matrix to which they bind by the emergence of new epitopes in the extracellular matrix that promotes angiogenesis, and via the switching of neoplastic cells to an angiogenic phenotype [26,65,66]. However, tumor-angiogenesis produce a tumor-associated vasculature that is chaotic, both in structure and function. These characteristics impair tumor blood flow and delivery of oxygen [26], but they favor its growth since without angiogenesis tumors rarely grows larger than 2 to 3 mm [65].

Hypoxia inducible factors drive the transcription of specific genes that promote tumor angiogenesis and systemic erythropoiesis [61]. Host tumors, which have grown larger than 1 mm^3 ^contain regions of low oxygen tension (hypoxia), making the formation of new blood vessels, or neoangiogenesis, essential for further tumor growth [66].

Tumor survival and metastasis are controlled by the balance between angiogenesis stimulators and inhibitors [50]. Among the angiogenic signals, vascular endothelial growth factor (VEGF) stands out [67,68]. VEGF also increases vascular permeability and stimulates the mobilization of endothelial progenitor cells [41,53]. HIF-1α mediates the expression of angiogenic proteins, and among them, VEGF is probably the most potent one [69,70]. In addition to upregulating angiogenesis, however, HIF-1 directly contributes to the downregulation of the oxidative phosphorylation and upregulation of glucose uptake and glycolytic enzyme expression [32,69,71].

The appearance of both local (tumor or T stage) and systemic (metastasis or M stage) angiogenesis would provide the tumor substances produced by the host, particularly hormones and growth factors, via the blood. This exogenous contribution, which the tumor cannot synthesize, could favor the expression and activity of glycolytic enzymes [32]. Initial studies found that a variety of oncogenes and growth factors could increase the expression of glycolytic enzymes [72]. Subsequently, the increased metabolic ability would increase the production of ATP, which would be used mainly for proliferation. Therefore, it is in this evolutive phase when cancer can be considered a growing disease [16,18].

The cancer cachexia syndrome, characterized by anorexia, weight loss with muscle wasting and increased energy expenditure [73-75], could be the result of an effective metabolic functional ability that the cancer cell develops to use the only available storage of substrates, those belonging to the host [18]. Hence, the mobilization of substrates that the SIRS produce in the host, through the alteration of the carbohydrate, lipid and protein metabolism with hypermetabolism, would favor its use by the cancer cell [76]. For this reason, it could be considered that in advanced stages of the cancerous disease, tumor cells acquire the ability to metabolically manipulate the host in order to benefit their own development [18].

Consequently, it could be considered that the successive phases of tumor evolution could have a trophic meaning, although the pathophysiological mechanisms involved would increase in complexity [16,18]. This hypothetical approach to the mechanism that governs tumoral evolution could be based on the increasingly metabolic capacity of the tumor cell to use oxygen over the successive phases of release, migration, and proliferation [77].

## Implication of the hypothesis

Since the phases of tumoral evolution, like the phases described of post-traumatic inflammation [11,13,14], go from ischemia to a progressive oxigenation, it is also tempting to speculate on whether the tumor cell reproduces some of the successive stages by which life passes from its origin without oxygen until it develops an effective, although costly, system for the use of oxygen [78,79]. If so, the successive metabolic switches that cancer undergoes allows it to acquire a growing ability, both to invade the host and use the host sources of substrates until its metabolic reserves are all used up [77].

The hypothesis that atmospheric oxygen concentrations affected the timing of the evolution of cellular compartmentalization by constraining the size of domains necessary for communications across membranes has been suggested [80]. Thus, the relatively rapid changes in the size of the oxygen-rich external domains coincide with increasingly organism complexity. This points towards a key role for oxygen in the increase in abundance and size of receptors over time [80] and adds to a growing body of literature connecting atmospheric oxygen levels with macroevolutionary changes, most recently with complexity in metabolic networks and cell types [78-80]. Therefore, a correlation between the increased organism complexity and the development of the use of the atmospheric oxygen could be established [79-81].

This correlation also seems to exist in the development and proliferation of malignant cells. Thus, it has been proposed that if cancer evolves in these three successive phases [16,18], progressive cellular complexity would occur parallel to a gradual oxygenation process. In this hypothetical situation, successive metabolic switches would be activated by tumor cells. Thus, an initial anoxic metabolism is gradually replaced by progressively more complex metabolisms characterized by a growing capacity to use oxygen [80].

Hence, during the first or nervous phase, the absence of (anaerobic phenotype) or low (reoxygenation phenotype) oxygen availability of the cancer cell is coupled with a decreased metabolic activity, low energy requirement and nutrition by diffusion. In this early stage, the tumor cell ha few surface membrane receptors, which in turn increase cancer cell autonomy and favors the autocrine function.

During the immune phase, cancerous cells acquire a leukocytic phenotype. The increased metabolic activity, associated with a greater use of oxygen, would favor the enzymatic digestive ability as well as an increased capacity for the intercellular or paracrine relation. Cancer cell derived paracrine signals may induce intercellular pathways that promote their leukocytic behavior with lymphatic migration and metastatic growth in lymph nodes.

In the last or endocrine phase, the tumor cell induces an angiogenic phenotype. The most primitive angiogenic tumor phenotype could be vasculogenic mimicry [82] or the novo generation of blood vessels, without the participation of endothelial cells. [82,83]. For this process, tumor cells acquire characteristics similar to endothelial cells, namely, they would be able to inhibit the above expressed leukocytic tumor phenotype (antioxidant and antienzimatic) and induce the abundant expression of adhesion molecules. This fact makes up an important component of the molecular switch to vasculogenic mimicry [84,85] to form a vascular-like tube formation [82,83,85]. Through this means, tumor cells contribute to conducting blood in vascular-like structures, a process that would be independent of regular angiogenesis [82,83,85].

By either vasculogenic mimicry [82,83] or by angiogenesis [53,65,68] the angiogenic switch promotes the endocrine function of the malignant tumor. In this phase, the cancer cell, through the angiogenic switch (endocrine), would be able to establish a metabolic activity in synergy with the host. Tumors, when vascularized, use substances, like neurotransmitters and hormones, synthesized by the host due to its (the host) correct functioning of oxidative phosphorylation [80]. These substances in turn could enhance the glycolytic activity of the tumor [30-32]. Therefore, the metabolic limit of the tumor in this evolutive phase is prevented by the additional contribution of mediators supported by the aerobic metabolism of the host.

As cancer cells develop mechanisms to create their vascularisation, their metabolic demands could increase, particularly those related with their excessive potential to proliferate, which finally cause a cachectic state in the host [73-75,86]. The malignant tumor thus causes a profound alteration in the metablolism of the host, adapting it for its own benefit. Thus, it increases oxidative stress, elevates resting energy metabolism, increases proteolysis with lean body mass wasting, enhances lipid mobilization and increases liver gluconeogenesis. In particular, neoglucogenesis allows the tumor cells a high contribution of its favorite substrate, that is glucose, for the anaerobic metabolism. [74,75,86].

During these evolutive phases, it could be considered that progressive metabolic tumor complexity is associated with a growing structural complexity. In particular, the use of the pathophysiological mechanisms of the inflammatory response to invade and take over the host would provide a considerable advantage for its development. In an attempt to create a increasingly more complex structure, the tumor cell is isolated from the neighbouring cells; it becomes autonomous (nervous phase), migrates (immune phase) and proliferates (endocrine phase) [16,18]. It could be speculated that due to the use of both, the tumor cell and the inflammatory response, of the same described basic mechanisms, they would also share the same objective, namely, the creation of new tissue. This neoforming capacity can be local (cicatrization), regional (regeneration) or systemic (embryogenesis) [13].

However, the cancerous cell is not able to reach the last basic mechanism that defines the inflammatory response, which is differentiation [12-14]. Perhaps, this functional incapacity of the malignant tumor cell is associated with the impossibility of expressing an efficient oxidative metabolism (oxidative phosphorylation) to obtain the large amount of energy that this specialized cell function requires. Due to this metabolic and functional limitation, the cancerous cell, although it recognizes potential targets of development during metastasis, is not able to differentiate.

The demonstration that the growth of tumors and metastasis are dependent on the development of their vascular supply [25,53,65-68,70] supports the idea that antiangionesis has been regarded as a target for cancer therapy [67,68,87,88].

However, a hypothetical therapeutic alternative would be to promote its transition from this stage of maximum aggressiveness, in which proliferation induced by angiogenesis predominates, to a more metabolically advanced stage that would induce its specialization. This last "step," which is normal in the post-traumatic inflammatory response [11-13], however, seems to be forbidden the cancerous cell. Perhaps, this limitation of the cancerous cell to become specialized, when it co-opts the inflammatory mechanisms of the host, is one of the main reasons why tumors are considered "wounds that do not heal" [89]. Hence, this would explain why the cancerous cell recognizes the chronic inflammatory processes to be the right environment for its development. [49,90]. In this hypothetical situation, the induction by gene therapy of the tumor cell specialization could be an alternative cancer therapeutic strategy. In essence, perhaps we only have to help the cell finish a process that was already initiated when metastatic colonization took place [53].

## Conclusion

The need to establish a link between the evolutive stages of cancer (TNM) and its biological significance, could be of interest for integrating the pathophysiological mechanisms that control this disease.

It has been hypothesized that while the malignant tumor develops, it can express phenotypes that also share the inflammatory response. These phenotypes can represent the expression of trophic functional systems of increasing metabolic complexity for using oxygen. Using the mechanisms characteristics of the inflammatory response, the tumor cell evolves through its release, migration and proliferation. The incapacity of tumor cell to successfully complete the last phase of the inflammatory response, namely, specialization, allows for considering new therapeutic alternatives based on its destruction.

## Competing interests

The author(s) declare that they have no competing interests.

## Authors' contributions

The three authors conceived, discussed and wrote the manuscript.
